# Rural research capacity: a co-created model for research success

**DOI:** 10.1186/s12961-023-01030-5

**Published:** 2023-07-24

**Authors:** Paige Farris, Rachel Crist, Sylvia Miller, Jackilen Shannon

**Affiliations:** 1 Community Outreach and Engagement Program, Knight Cancer Institute, Oregon Health and Science University, Portland, OR 97239 United States of America; 2 Oregon Clinical and Translational Research Institute, Oregon Health and Science University, Portland, OR 97239 United States of America; 3 Present Address: Division of Oncologic Sciences, Knight Cancer Institute, Oregon Health and Science University, 3181 SW Sam Jackson Park Rd., Portland, OR 97239 United States of America

**Keywords:** Rural, Clinical research, Implementation, Community, Cancer prevention and control

## Abstract

**Purpose:**

The United States’ National Institutes of Health (NIH) have long challenged academia to improve clinical trial enrollment, especially in underrepresented populations; inclusive of geography, age, disability status, racial and ethnic minorities. It has been shown that rural and urban residents enrolled in clinical trials have similar outcomes, yet, rural healthcare systems struggle to provide opportunities to rural residents to participate in clinical trials when infrastructure is limited or unsupportive of research programs and/or research staffing levels are insufficient. To fully address the barriers to clinical trial access in rural areas, it is not adequate to simply open more trials. Community receptivity of research as well as organizational and community capacity must be considered. This project was determined by the Oregon Health and Science University’s Institutional Review Board to be generalizable research across the chosen counties and was approved to operate under a waiver of written consent. Participants received a cash incentive in appreciation for their time and verbally agreed to participate after reviewing a project information sheet.

**Methods:**

The research team co-created a community-responsive approach to the receipt, review, and acceptance of clinical trials in a rural community setting. An adapted 5 step Implementation Mapping approach was used to develop a systematic strategy intended to increase the success, and therefore, the number of clinical trials offered in a rural community.

**Results:**

The research team and participating rural community members pilot-tested the implementation of a co-designed research review strategy, inclusive of a Regional Cultural Landscape and three co-created project submission and feasibility review forms, with a cancer early detection clinical trial. The proposed clinical trial required engagement from primary care and oncology. Utilizing the research review strategy demonstrated strong researcher-community stakeholder communication and negotiation, which resulted in early identification and resolution of potential barriers, hiring a local clinical research coordinator, and timely trial opening.

**Conclusion:**

To the knowledge of the research team, the work described is the first to use a community-engaged approach for creating a clinical trial implementation strategy directly supportive of rural-sitting community stakeholders in receiving, reviewing, and approving cancer-related clinical trials in their community. Participating community members and leaders had the chance to negotiate research protocol changes or considerations directly with researchers interested in conducting a cancer clinical trial in their rural setting.

**Supplementary Information:**

The online version contains supplementary material available at 10.1186/s12961-023-01030-5.

## Introduction

Residents of rural regions of the United States suffer disproportionally from cancer as compared to their urban counterparts [1]. Clinical trial participation is a strong and consistent predictor of more positive cancer outcomes, and when considering only those who were enrolled in clinical trials, rural and urban residents experienced similar cancer outcomes [2]. Yet, clinical trial accessibility for rural patients continues to be low [3] and for a variety of reasons, even when accessible, clinical trial participation among residents of rural regions is lower than among urban residents. The United States’ (U.S.) National Institutes of Health (NIH) revised the definition of ‘clinical trials’ in 2014 to include the full continuum of research studies; from minimal risk observational studies, *cancer prevention and control research*, to potential high-risk Phase I, II, II drug-testing, efficacy, and safety studies [4]. Within every National Comprehensive Cancer Network (NCCN) guideline, there is the following statement: “Clinical Trials: NCCN believes that the best management of any patient with cancer is in a clinical trial. Participation in clinical trials is especially encouraged.”; as a standard of care, all cancer patients should be offered the opportunity to participate in a clinical trial [5]. The U.S.’s National Institutes of Health (NIH) have long challenged academia to improve clinical trial enrollment, especially in underrepresented populations; inclusive of geography, age, disability status, racial, and ethnic minorities.

A recent scoping review from McPhee et al. [6] suggests that if given the opportunity, rural residents will participate in clinical trials. Yet, there remain substantial barriers to this participation, including structural and clinical barriers as well physician barriers such as discouragement from their oncologist or primary care physician and patient barriers including monetary burden, length of commute, and lack of information [7]. In this same study by Virani et al., they suggest that rural patients are more likely to participate in cancer prevention/screening trials than therapeutic/treatment trials [7]. Rural residents may find prevention/screening trials more appealing as they may often be able to enroll locally and not require substantial travel costs and time. Although, per the findings of this article’s research team, prevention/screening trials or community interventions are not consciously aligned with primary care, regional health improvement priorities, home health, or other creative, intact methods for offering prevention/screening opportunities through locally-identified resources, assets, or work. Leveraging the limited resources within rural and frontier regions and assuring there is multi-level involvement of regional assets may improve the currently limited implementation of prevention/screening trials [8].

Efforts to increase participation of rural communities in clinical trials are growing, but have focused primarily on structural barriers, defined by Unger et al., as factors impacting trial availability [9]. Additional efforts focused on physician and patient barriers have ranged from efforts to increase provider referrals [10] to working with community organizations, faith-based organizations and community health workers to encourage trial participation [11]. While this work is important and a necessary component of trial success, it does not address how to encourage bidirectional or even wide promotional communication between rural communities and researchers such that the needs of the community may be brought forward and addressed before a clinical trial is initiated. Communication and direct collaboration, if it happens, is often inconsistent and may be between one individual in a community or clinic setting and the researcher; which may not reflect the realities of the overall, unique community, and therefore, a larger population of eligible participants.

A systematic strategy that sits within and is directed by Oregon communities for reviewing, vetting, and implementing purposeful bidirectional communication and which offers legitimized negotiation powers between rural communities and academician researchers is needed. To the research team’s knowledge, there is not an existing structure for introducing such trials, increasing primary care comfort and knowledge about promoting clinical trials (other than increasing education directly with providers), nor how to align non-interventional research with primary care’s daily patient healthcare processes [12–15]. Partnerships help foster new processes and structures to facilitate clinical trial participation in their communities and patients who receive information about clinical trials from their healthcare provider were significantly more likely to participate in clinical trials [11, 15–19]. The U.S. NIH’s National Cancer Institute (NCI) offered NCI-designated cancer centers an opportunity to respond to this complex challenge and specifically learn about the barriers and facilitators to conducting cancer prevention and control (CPC) trials in rural areas. In 2019, the institution participating in this project joined a second round of NCI Rural Cancer Control grant recipients, with the specific goal of co-creating a mechanism to engage and support rural cancer centers in bringing CPC trials to their patients. Two researchers and a Community Research Liaison from the region, a local community member working to facilitate relationship between this institution’s CPC trial efforts and the Liaison’s community, led this work together with members of two rural counties in the state. Kenny et al. [20] recommended and cited rural community engagement concepts that the research team followed and implemented throughout this project. Rural areas are not carbon copies of one another and engagement in each rural area in Oregon is required in order to respond to the intricacies of multi-levels of influence, healthcare [20], and clinical trials access. This work not only expands upon what others have noted but offers the ‘processes’ through which the research team came to co-develop a foundational system for receiving rural community feedback to incoming clinical trial research, which had not been listed in previously conducted work. Specifically, the research team approached this project with perspectives described by Kenny et al. [20]. Planners recruited community champions with a creative and inclusive eye about connections across the region. The team not only engaged with primary care physicians and oncologists in relation to vetting CPC trials but also with librarians who help community members conduct research on disease, home health providers who offer care in rural residents’ homes, community health workers who have deep, trusted relationships within the community, with long-standing research program managers in a rural cancer center, etc. This effort was not ‘symbolic’[20]. In order to address barriers to developing sustained opportunities for CPC trial participation in rural regions, a multi-level, multi-factorial community engagement framework was applied (e.g., the socioecological model) [21]. The research team also approached this project with a humble acknowledgment that community members are experts in and about their own communities [22–24] and that each level of influence in a community is interrelated with all other levels. The research team’s community engagement approach is reflective of Dr. Gil Friedell’s (the first director of the University of Kentucky’s Markey Cancer Center) perspective; “if the problems are in the community, the solutions are in the community” [25]. Per studies conducted by others [26–28], the research team added cultural context to the needs assessment step, requested input to the proposed clinical trial by rural communities as well as offered authentic, tangible opportunities to partner with incoming researchers to local/non-clinical adopters, implementers, influencers, and champions.

The goal of this study was both to acknowledge regional limitations to CPC trial participation, and to work collaboratively with rural community partners to develop a structured process for reviewing and introducing studies that integrates known community and clinic system limitations into capacity and feasibility assessments of incoming CPC trials. By utilizing the co-developed process, a local cancer center works collaboratively with primary care providers, local community stakeholders and researchers to identify trials most likely to be successful, and then collaboratively lays the groundwork for trial implementation.

## Methods

The overall goal of this minimal risk project was to implement a community-engaged approach to co-create a tool that would help guide researchers to offer CPC trial opportunities in rural regions. Specifically, we aimed to develop a systematic strategy intended to increase the number and success of CPC trials in a rural community. To this end, the work was not guided by a specific research question, but was intended to develop a process that could support the successful conduct of research.

**Fig. 1:**
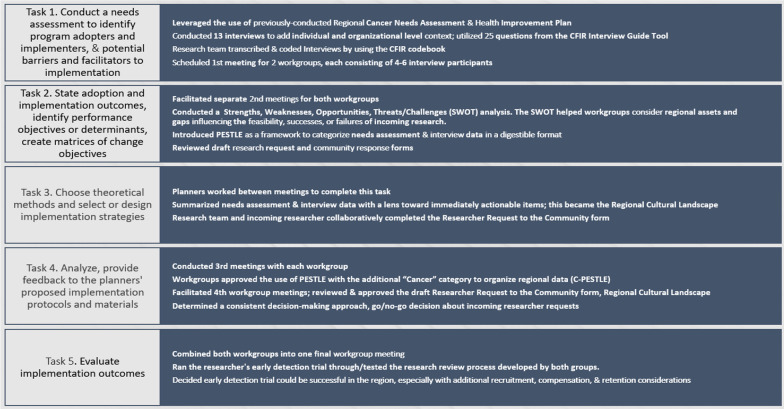
Implementation mapping steps and research team’s methods

The research team used Implementation Mapping as the primary framework for this community engaged project. Implementation Mapping is a 5-step process for guiding the development of an implementation strategy, using theory, evidence, and stakeholder input [29]. The research team acted in the role of implementation planners. The Consolidated Framework for Implementation Research (CFIR)[30] was a tool used to help describe contextual factors and the research team chose interview questions that focused on the umbrella domains of the regional intervention characteristics, outer settings, inner settings, characteristics of individuals, and process implementation (see Additional file 1: Interview Questions]). Utilizing CFIR questions helped the research team understand regional contextual realities that would influence adoption and implementation of a co-created system [29]. Implementation Mapping steps (tasks) include (1) conduct a needs assessment to identify program adopters and implementers, as well as potential barriers and facilitators to implementation, (2) state adoption and implementation outcomes and performance objectives, identify determinants and create matrices of change objectives, (3) choose theoretical methods and select or design implementation strategies, (4) produce implementation protocols and materials, and (5) evaluate implementation outcomes. The implementation planners walked two workgroups (4–6 participants each) through the 5-step process by facilitating and scheduling meetings, developing products based on feedback from participants, and assigning homework to participants between workgroup meetings (Fig. 1).

Implementation Mapping Task 1. Conduct a needs assessment to identify program adopters and implementers, and potential barriers and facilitators to implementation. The research team leveraged and referenced local, previously conducted community health needs assessments relevant to the rural region. Needs assessments are regularly conducted at hospital, community, county, and sometimes regional levels throughout Oregon in response to state-level funding or hospital accreditation requirements. Data was identified and aggregated from existing county and/or regional health assessments as well as from a cancer needs assessment conducted by this team’s academic NCI-funded cancer institute. The cancer needs assessment utilized publicly available state level data, to document cancer-related needs in the regional cancer center’s catchment area. This publicly-available data includes regional cancer incidence and mortality rates, cancer prevention metrics, and modifiable risk factors to inform the development of a clinical trial implementation process.

To collect an assessment of individual and organizational level barriers and facilitators to and gaps or assets in support of conducting CPC trials instead of relying solely on aggregate regional data for this implementation mapping process, the research team conducted 13 interviews, some lasting up to three hours over two sessions, with individuals who could offer perspectives from the county, community, hospital, clinic, provider, and patient levels (see Table 1). Interviews took place over five months in 2020–21. To identify participants, the research team leveraged one of five regional Community Research Liaisons who are also employed on the overall team.

**Table 1:** Demographics of participants (n = 13)

	n	%
Gender		
Female	6	46
Male	7	54
Age range		
25–49	7	54
50 plus	6	46
Race	White	77
	Asian	15
Ethnicity	Hispanic	8
	Non-Hispanic	92
Level of Education	Bachelor's	31
	Master's	46
	Doctorate	23
Length of time in position or current setting		
	1–2 years	23
	3–4 years	31
	7–10 years	10
	> 10 years	38

Community Research Liaisons live and work in rural areas of the state. Community Research Liaisons bring a depth of regional knowledge and personal connections to projects that are difficult to develop from academic institutions located in, generally, metro areas. The Community Research Liaison utilized a snowball approach to interview recruitment and to identify potential adopters, implementers, maintainers and other leaders who could best respond about regional assets and gaps. The Institutional Review Board determined that this public health, minimal risk research project was generalizable in our chosen counties, employed social science methodology, and was approved to operate under a waiver of written consent. Each participant received a study information sheet that described the purpose of the project, participants had opportunities to ask questions at the time of initial study procedure (e.g., recruitment contact, interviews) as well as throughout the conduct of the study, was verbally consented to the project, and compensated for their time.

The initial set of 52 (not inclusive of sub-questions) CFIR interview questions were collected utilizing the CFIR Guide Interview Guide tool; linking to the ‘Choose Interview Questions’ [31] site and were reduced to 25 based on expert review from the local Community Research Liaison and the research team lead. These questions focused on gaining participant perspectives on (1) the compatibility of their organization’s priorities with cancer control trials; (2) their organization’s openness to the implementation of cancer control trials based on organizational culture, internal champions, and opinion leaders as well as outside pressures; and (3) needs of the community members served by their organization. All interviews were video or audio recorded and members of the research team (*n* = 3) took their own notes to capture quotes, ideas, and perspectives of relevance to the project.

Interview recordings were transcribed and coded by the research team using the CFIR codebook and CFIR codebook spreadsheet documenting data analysis prompts, inclusive of detailed definitions about, inclusion and exclusion criteria for CFIR domains and descriptions of domain subitems [32]. The three research team members co-coded 1–2 of the transcripts in order to support the team’s collective understanding of the codes and assure intercoder reliability. The transcripts were analyzed according to the CFIR domains (Table 2) of inner setting, outer setting, individual characteristics, and process implementation.

**Table 2:** CFIR constructs and interview question categories

Inner setting (Community Organizations*)	Outer setting (Community/Region wide)	Individual characteristics	Process implementation
• Structural Characteristics • Goals and Feedback • Compatibility • Culture • Relative Priority • Implementation Climate • Tension for Change	• Cosmopolitanism • Peer Pressure • Patron Needs, Resources and Barriers	• Knowledge and Beliefs about the Intervention • Adopters and Implementers	• Engaging Opinion Leaders • Engaging Champions

*Community Organizations represented by participants included a public health department, Federally-Qualified Health Center, libraries, a hospital cancer center, primary care medical center, a certified rural primary care clinic, a K-12 school district, an extension office, and home health

Interviews were used to identify adopters and implementers in the region. The research team invited all interviewees to participate in two community workgroups consisting of 4–6 participants each. The research team (planners) referenced Implementation Mapping tasks 1–5 to organize workgroup meetings and outcomes. Workgroups met virtually and a virtual whiteboard platform was used to create engagement and capture participant responses, feedback, and input. Both workgroup meetings had the same agenda items, activities to cover, and received a reminder email a week in advance. Each workgroup met four times separately and once as a larger group over the course of a month and a half. Between each meeting, the research team compiled workgroup ideas and feedback into documents in order to iteratively develop a research review process. In each reminder email, participants were asked to review the documents and consider the proposed processes.

During the first workgroup meeting, Task 1 questions were used to guide community member activities and identify who the adopters, implementers and maintainers of the “intervention” (specifically, the process being collaboratively developed to support CPC trials) might be in their community. The research team specifically requested input about;

*Who will decide to adopt and use the process we develop? Which stakeholders will decision makers need to consult? Who will make resources available to implement the process we develop and CPC trials? Who will implement the process we develop and CPC trials? Will CPC trials require different people to implement different components (and should those people be involved in the working group? And who will ensure that the process we develop continues as long as it is needed?*

Despite relying heavily on the CFIR framework and codebook, eventual themes did not fully describe the community context which organizations operate within and how CPC trials could be implemented, leading to a later step of creating a researcher reference tool (see Additional file 1: Regional Cultural Landscape), based on a non-CFIR framework (described as part of Implementation Mapping Task 3, below), to better share a deeper community context with researchers.

Implementation Mapping Task 2. State adoption and implementation outcomes, identify performance objectives/determinants, create matrices of change objectives. During the second workgroup meeting, a mini strategic Strengths, Weaknesses, Opportunities, Threats/Challenges (SWOT) analysis was introduced as a method for organizing researcher requests as strengths and weaknesses against the opportunities and challenges in the region that had been identified from thematic analysis [33]. The research team also introduced, conceptually, a PESTLE Analysis as a framework to organize the broad, in-depth needs assessment that had been iteratively collected throughout this project [34]. The use of PESTLE in relation to Implementation Mapping Task 3 will be discussed as a step included in the implementation strategy. The goal of the SWOT process was to identify a potential approach and scoring mechanism for how well incoming research requests could meet and leverage the assets and fill in gaps of the region, meet the region’s ability to support a project, and which would lead to successful or feasible implementation of CPC trials [33].

Draft forms were reviewed during the second workgroup meeting for input to the question; “*What has to change in order to bring about the performance objective (implementation of CPC trials in the region)?*” [29] Workgroup members reviewed the draft forms in the context of their own lived experiences, knowledge about regional resources, needs assessment data and contributed ideas in the online white board space.

Implementation Mapping Task 3. Choose theoretical methods and select or design implementation strategies. Task 3 of Implementation Mapping, prioritizes developing and/or selecting strategies that align with the previous steps taken by researchers[35, 36]. Workgroup members approved the use of the PESTLE, adding ‘C’ to represent cancer-related information, to best organize regional data collected both through interviews and referencing previously conducted assessments. Planners organized the needs assessment data with a lens toward immediately actionable items and input [29]. The eventual product inclusive of all data collected, organized, and cultivated became the *Regional Cultural Landscape* [35].

Implementation Mapping Task 4. Analyze, provide feedback to the planners’ proposed implementation protocols and materials. During the third workgroup meetings, the research team presented the organized needs assessment data (cancer rates, cancer prevention metrics, modifiable risk factors and thematic analysis results) into a densely populated C-PESTLE slide deck. The C-PESTLE informed discussion around two specific questions:

A) what information about the community, patient population, and region (e.g. the cultural context within which partnering community organizations work) would be useful for researchers to understand and respond to prior to initiating a clinical trial, and
B) what resources, assets and processes are necessary for clinicians, the hospital, other clinical settings, CPC-aligned community-based organizations and community members to consider in assessing the feasible implementation and collaborative initiation of a regionally-conducted clinical trial.

The dense C-PESTLE was reduced to a two-page *Regional Cultural Landscape*, without omitting main priorities, important considerations, or communities’ cultural context; encouraging researcher digestibility. Each workgroup was asked to review all products developed and assure relevant prompts were in place for helping the community to decide whether a project could be run successfully or not.

Implementation Mapping Task 5. Evaluate implementation outcomes. The final step of the Implementation Mapping process was addressed during the fourth and fifth workgroup meetings. Prior to the fourth workgroup meeting, the research team met with a researcher, providing the *Regional Cultural Landscape* for review and the *Researcher Request to Community* form (Request form) for completion. Collaborative completion of this form by the research team and the researcher took approximately one hour.

Members of both workgroups were brought together for the fifth and final workgroup meeting to conduct an overview of the research review process that was developed by both groups in previous meetings, to determine a consistent decision-making approach, and to determine a regional home to the research review process by identifying potential adopters, implementers, and maintainers within the community.

**Fig. 2:**
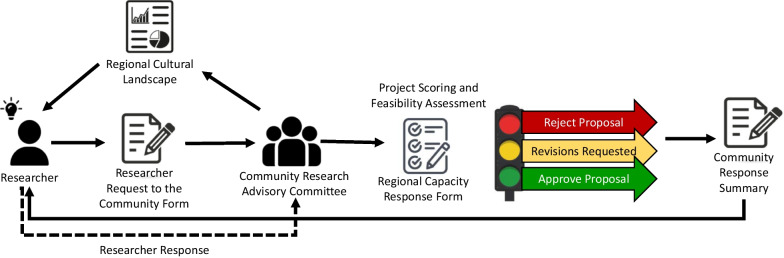
Final Research in Oregon Communities’ Review System (ROCRS)

The final step in this implementation project was to follow the co-developed research review process with an incoming research request/clinical trial. Length of time necessary for each step in the process, who completed steps (e.g., adopters or implementers), and where additional support may be necessary was captured. An industry-sponsored early detection trial, run by an investigator at this team’s cancer institute, was identified as appropriate for testing the process as the trial requires input from clinics and providers across specialties and recruits from a largely healthy population. Primary care clinicians participate at the time of patient recruitment and oncologists are engaged at the time of testing follow up. See Fig. 2 for a visual representation of the full process.

Completion of the research review process and approval of the project took 3 weeks; an additional 6 weeks were required for the rural cancer center to complete the non-disclosure agreement and identify a “sub-investigator” oncologist. From initiating the review process, to receipt of academic Institutional Review Board (IRB) approval to add the rural cancer center as a site, hiring a local research coordinator and recruiting the trial’s first research subject, it took 6 months. In comparison, at the research study’s academic institution, the average time from an investigator accepting participation in a clinical trial, completion of contracting, IRB submission and approval, to recruitment of first subject also takes about 6 months. Thus, this research review process does not add substantially to start up time, and importantly, it assures community input, interest and involvement with researchers about an incoming clinical trial, the early identification of stakeholders and a pro-active, informed approach to addressing possible barriers to success.

## Results

Implementation Mapping Task 1. Conduct a needs assessment to identify program adopters and implementers, and potential barriers and facilitators to implementation. In addition to utilizing previously-conducted regional health needs assessment data, qualitative analysis was conducted on the interviews. Emergent codes were identified within the broader categories pulled from CFIR [32] and interview notes, while additional codes unique to the location and questions, such as “small town,” and “provider landscape” were added. While identifying assets and gaps that may support and hinder the conduct of clinical trials in the region, details such as “complexity of resource needs,” identify natural opportunities, locations or fit for CPC clinical trial implementation and specific approaches to engaging in the community were coded. The coded themes (Table 3) showed commonalities across;.

- regional challenges,
- opportunities,
- community-wide characteristics (community members and providers),
- actionable guidance (e.g., engagement strategies, how to/not to engage with participants),
- organizational alignment with CPC work and
- community member needs.

**Table 3:** Thematic findings from interviews

Inner setting (Community Organizations)	Outer Setting (Community/Region wide)	Individual characteristics	Process implementation
Adopters Implementation Climate: • Hard • Easy • Engagement Strategies How to Engage, How Not to Engage with Participants, Engagement Considerations • Resource Need Opportunities • Resource Need Challenges Maintenance and Sustainability • Who/Entities • How to Align Cancer Prevention and Control Research with Current Organizational Priorities • Available Resources Decision-Makers/Entities Conducting Cancer Prevention and Control-Related Work Currently • Challenges	Community wide: • Fierce Independence • Characteristics/Culture-Opportunities • Characteristics/Culture-Challenges • Resource Needs-Opportunities • Resource Needs-Challenges Provider Landscape: • Recruitment and Retention • Fierce Independence • Opportunities Small Town: • Resource Need Challenges	People/Implementers for Different Cancer Prevention and Control Components	NOTE: the research team determined there were no codes that fit this CFIR category since 1) The research institution had conducted needs assessment interviews at the community organization level, 2) these community organizations were not to be the ‘host’ of the research review process, and 3) an implementation system had not yet been created


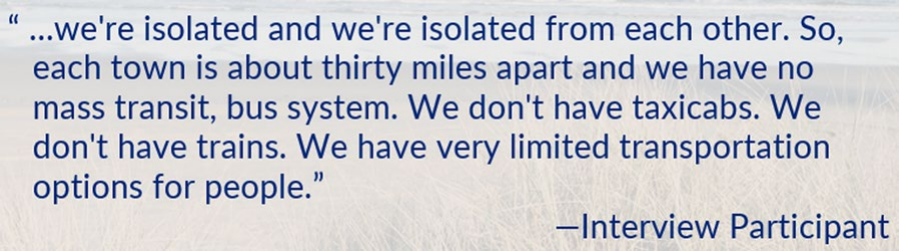


**Fig. 3:**
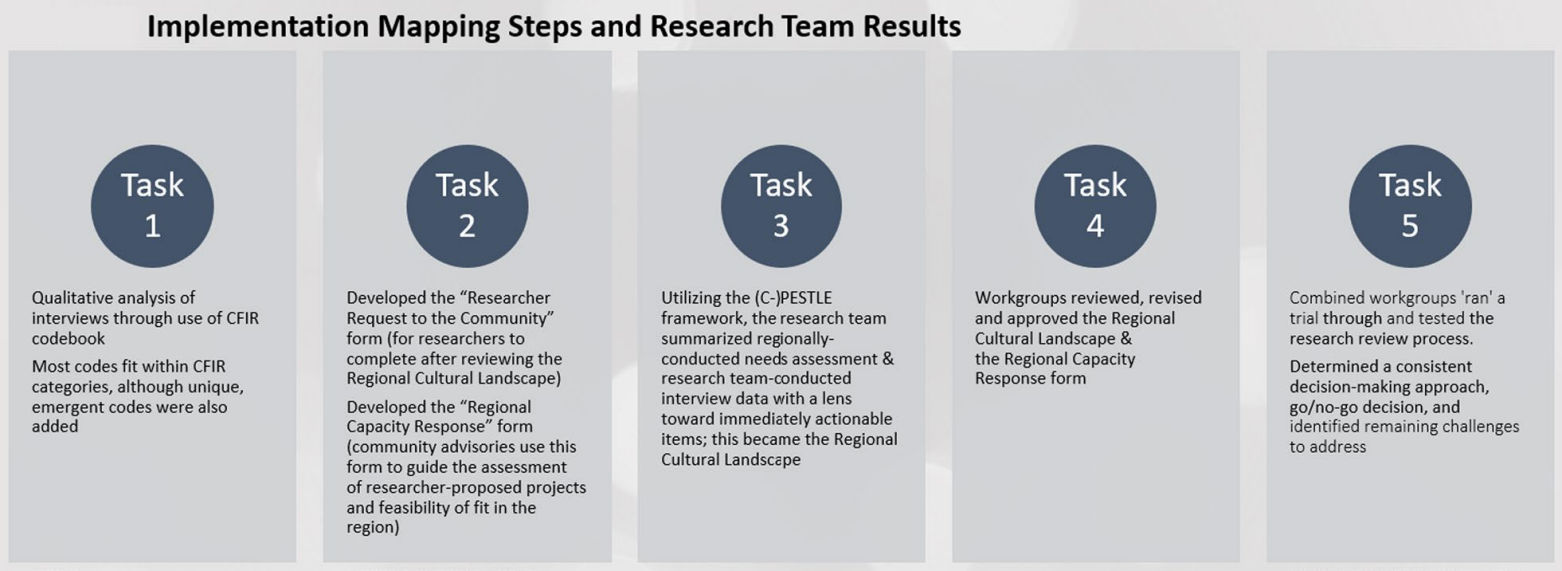
Implementation mapping steps and research team results

Implementation Mapping Task 2. State adoption and implementation outcomes, identify performance objectives/determinants, create matrices of change objectives. Based on the prompts of a SWOT scoring matrix, the research team developed a draft “*Researcher Request to the Community*” form and “*Regional Capacity Response*” form (see Appendix documents 3 and 4). The *Researcher Request to Community* form was adapted from an existing site assessment questionnaire developed to assess rural cancer centers’ ability to conduct clinical trials. The research team retained open and closed ended questions about assets and gaps a clinical trial might be able to fill (e.g., strengths and weaknesses of the mini-SWOT analysis), and included questions specific to how a proposed trial could acknowledge and be consciously responsive to stated needs about the community of interest (e.g., opportunities and challenges of the mini-SWOT analysis). For instance, as the quote from an interviewee states, transportation is a major hurdle in the region and is an issue that researchers bringing their studies into the region should be conscientious of and responsive to if they wish their trials to be successful. The *Regional Capacity Response* form was adapted from existing clinical trial feasibility assessments also currently in use by the research team’s cancer institute. The purpose of this form is to assess fit between an incoming research request and the region’s ability to support the specific project request. Figure 3 describes the research team's results in relation to each implementation mapping step (Fig. 3).

**Fig. 4:**
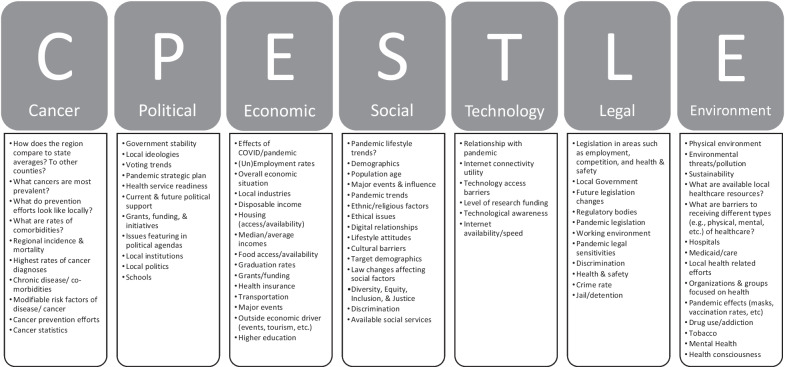
Adapted PESTLE analysis framework

Implementation Mapping Task 3. Choose theoretical methods and select or design implementation strategies. In following with the previous Implementation Mapping steps, the research team organized all needs assessment data into a standardized format that included information about the political, economic, social, technological, legal, and environmental characteristics of the region, adding a ‘C’ to represent ‘cancer’ (C-PESTLE see Fig. 4). This strategy allowed for the team to present the collected data in a way that would be digestible to community members as well as to researchers that “*preserves the parameters for effectiveness and fits with the target population, culture, and context”* [35].

Implementation Mapping Task 4. Analyze, provide feedback to the planners’ proposed implementation protocols and materials. To address the question; *what information about the community, patient population, and region (e.g. the cultural context within which partnering community organizations work) would be useful for researchers to understand and respond to prior to initiating a study*, each workgroup reviewed the C-PESTLE, contributed additional contextual perspectives, requested additions and refinements and also proposed developing a more easy-to-digest summary of the region for researchers to reference; the *Regional Cultural Landscape*. To address the second question; *what resources, assets and processes are necessary for clinicians, the hospital, other clinical settings, CPC-aligned community-based organizations and community members to consider in assessing the feasible implementation and collaborative initiation of a regionally-conducted clinical trial*, the workgroups reviewed the revised *Regional Capacity Response* form to assure it addressed the areas identified as needs in the initial assessment, adding 13 prompts in three different sections (Population of Interest [*n* = 2], Project Plan/Protocol [*n* = 2], Budget and/or Resource Needs [*n* = 9]). As a reminder, the *Regional Capacity Response* form is meant to align the clinical trial (the Strengths and Weaknesses potentially impacting a region) against Opportunities & Threats/Challenges (e.g., facilitators and barriers) relevant to and identified in the region (per mini-SWOT and all collected data). This form therefore includes prompts the community requires for deciding whether a trial can be successfully run or not.

Implementation Mapping Task 5. Evaluate implementation outcomes. During the fourth workgroup meeting, the research team facilitated a discussion and documented input or questions directly within the *Regional Capacity Response* form (Response form) and incoming clinical trial scoring. The Response form contains 35 questions that the community considers in their decision-making process; 30 yes/no questions and 5 narrative responses or opportunities to ask for researcher clarification/additional information as well as one open text field available to ask for detail about trial-specific questions. Workgroups were enthusiastic about the cancer early detection trial and believed it would be well-received among both clinical and patient communities, giving the researcher a yellow light to initiate the trial. The main highlights of concern included conduct of the clinical trial by a local research coordinator, transportation support, and the researcher’s acknowledgment of the severe lack of provider capacity in the region, that is, doing as much to avoid additional provider burden as possible. Of 38 specific questions the community sent back to the researcher for more information, the researcher directly answered 19. Additionally, the researcher was willing to negotiate different points with the community, including considering transportation reimbursement, offering a $10 incentive per survey completion, willingness to meet/promote the trial in local clinics, electronic medical record linkages to alleviate provider burden, consideration of marketing and internal hospital community engagement promotion. The community suggested additional promotional avenues (via local influencers) and leveraging people in the community, like case managers, traditional health workers, caregivers, the disabled community, and to identify methods for reaching out to the houseless community. The researcher did not leverage these workforces for promotion nor suggestions for accessing differently-abled community members.

The research team directly connected the researcher to relevant community members and the researcher initiated and completed (1) hiring a full time, locally-residing research coordinator to support trial implementation on the ground, (2) a non-disclosure agreement with the local cancer center, and (3) recruiting a cancer center’s oncologist to act as a ‘sub-investigator’ to the academic cancer center-led project.

Workgroup members identified that the research review process needed to overcome several existing challenges, including the inconsistent frequency of research requests and the lack of additional capacity to support a new committee in the region. Taking these factors into consideration, the workgroup proposed a process that could be “plugged into” existing groups and standing meetings. Further, it was important to determine a consistent decision-making approach. The combined workgroup panel supported the implementation of a “stoplight” approach; red for rejections where projects had too many barriers to be successful in the community, yellow for when barriers were identified but the advisory committee saw potential in the trial if the researcher could effectively address barriers, and green for when there were few to no barriers present for a project to be feasible and potentially successful in the community. This process was developed to ensure consistent and sustained use of all developed materials reflective of the community and its needs; the *Regional Cultural Landscape*, the *Researcher Request to the Community* and the *Regional Capacity Response* forms.

After finalizing the research review process, the workgroup helped craft an early version of what would later become the *Community Response Summary,* detailing community feedback, summary of their discussion, project strengths and barriers to success identified by the community, and their position about whether or not to initiate the trial. The *Community Response Summary* would later be formalized to summarize the advisory’s discussion and *Regional Capacity Response* form, outlining the (1) top three to five strengths of a project; (2) top three to five barriers to address to move a project into “Green Light”; (3) community recommendations; and (4) initial Red, Yellow, Green response from the community about the presented project.

## Discussion

Previous studies have shown that cancer patients living in rural regions experience worse outcomes than their urban counterparts. Additionally, rural residents have less local access to academician-directed research or clinical trial opportunities, partially due to being under-resourced. While a number of studies have identified reasons for lower trial participation among rural residents [7], and there have been attempts to increase rural provider participation in the promotion of clinical trials [37], little work has focused on the rural community as a whole and the need to include the community voice in decision making about implementation of trials [27]. This research team embarked on a collaborative process to co-create a research review system that was community-defined, represented the unique cultural context of a rural Oregon region, while also developing researcher and clinical trial paths.

An Implementation Mapping approach to guide this work with rural community stakeholders was chosen as it provided a more structured and defined CFIR methodology for the research team to follow. The Implementation Mapping process was an iterative approach to co-creating a research assessment strategy (the “intervention”) responsive to a rural, resource-limited region. Methodical movement through Implementation Mapping tasks allowed for the resulting process to be initiated to fidelity alongside community-based organizations, other clinical settings, and research-interested individuals in the area [29]. The research team (the “planners”) facilitated a multi-level informed community-engaged process to bring research to this rural region and assured that the planners were responsible for the burden of the work. The research team organized meeting agendas, proposed models for aggregating data (e.g., C-PESTLE), the research review system and created materials based on community input, previously-conducted health needs assessments, and lived experiences of the engaged workgroup members. All activities were supportive, not directive, and the final system is sustained with and by the community, and facilitated by the regional Community Research Liaison (Additional file 1: Regional Cultural Landscape, Researcher Request to Community form, Regional Capacity Response form).

## Limitations

The work reported here describes a novel, community-engaged process for introducing, reviewing and supporting CPC trials in the community but it is important to note a few limitations. “Rural contexts vary greatly from urban contexts and each other.”(Kilpatrick, 2009). This research review system has been fully implemented in only one community, potentially limiting generalizability to other Oregon communities. However, through the use of a replicable, methodologic approach and a willingness to adapt the process based on local context and guidance, this system may be effectively implemented in other communities to support feasible and community-responsive clinical trial implementation. The number of people who participated in the two regional workgroups was somewhat limited (4–6 per workgroup). While ideally there would be up to 10 individuals per workgroup, the size reflects, simply, the size of the community within which the project was conducted, and, second, the unfortunate impact of the COVID-19 pandemic that required a virtual approach to meetings and, therefore, limited the availability of clinical providers and other informants.

## Conclusions

To the knowledge of the research team, the work described here is the first to use a community-engaged approach for creating a research implementation strategy directly supportive of community stakeholders in receiving, reviewing and approving cancer-related clinical trials in their community. In future implementation and adaptation throughout Oregon’s rural regions, communities will be utilizing—and have already understood the practicality of utilizing—this research review system with a range of clinical trials, observational research, and community-driven studies, efforts, or priorities. This approach, which expands upon the idea of a research feasibility assessment, provides a community-responsive mechanism for researchers to learn from and about communities of interest prior to introducing a clinical trial. This information, defined here as the community’s cultural landscape, can adversely affect or importantly impact the success of a proposed clinical trial. When researchers thoughtfully consider and respond to community norms, feedback, assets and gaps, this active acknowledgment can influence the success of and a community-level endorsement of incoming studies.

## Supplementary Information

**Additional file 1:** Appendices: Interview guide and ROCR system products.

## Data Availability

The qualitative dataset generated and analyzed during the current study is not publicly available due to the fact that they constitute non-generalizable research data. However, the thematic analyses and the tools developed as part of this work are available from the corresponding author upon reasonable request.

## References

[1] Henley SJ, Anderson RN, Thomas CC, Massetti GM, Peaker B, Richardson LC (2017). Invasive cancer incidence, 2004–2013, and deaths, 2006–2015, in nonmetropolitan and metropolitan counties—United States. MMWR Surveill Summ.

[2] Unger JM, Moseley A, Symington B, Chavez-MacGregor M, Ramsey SD, Hershman DL (2018). Geographic distribution and survival outcomes for rural patients with cancer treated in clinical trials. JAMA Netw Open.

[3] Mudaranthakam DP, Gajewski B, Krebill H, Coulter J, Springer M, Calhoun E, Hughes D, Mayo M, Doolittle G (2022). Barriers to clinical trial participation: comparative study between rural and urban participants. JMIR Cancer.

[4] Health NIO. NIH's definition of a clinical trial. 2014. https://grants.nih.gov/policy/clinical-trials/definition.htm. Accessed 15 Oct 2021.

[5] Mohler J, Bahnson RR, Boston B, Busby JE, D'Amico A, Eastham JA, Walsh PC (2010). NCCN clinical practice guidelines in oncology: prostate cancer. J Natl Compr Canc Netw.

[6] McPhee NJ, Nightingale CE, Harris SJ, Segelov E, Ristevski E (2022). Barriers and enablers to cancer clinical trial participation and initiatives to improve opportunities for rural cancer patients: a scoping review. Clin Trials.

[7] Virani S, Burke L, Remick SC, Abraham J (2011). Barriers to recruitment of rural patients in cancer clinical trials. J Oncol Pract.

[8] Kennedy AE, Vanderpool RC, Croyle RT, Srinivasan S (2018). An overview of the National Cancer Institute's initiatives to accelerate rural cancer control research. Cancer Epidemiol Biomarkers Prev.

[9] Unger JM, Vaidya R, Hershman DL, Minasian LM, Fleury ME (2019). Systematic review and meta-analysis of the magnitude of structural, clinical, and physician and patient barriers to cancer clinical trial participation. J Natl Cancer Inst.

[10] ASOC Oncology (2009). Enhancing clinical trial awareness and outreach. J Oncol Pract.

[11] Baer AR, Michaels M, Good MJ, Schapira L (2012). Engaging referring physicians in the clinical trial process. J Oncol Pract.

[12] Bylund CL, Michaels M, Weiss ES, Patel S, D'Agostino TA, Binz-Scharf MC, McKee D (2021). The impact of an online training program about cancer clinical trials on primary care physicians' knowledge, attitudes and beliefs, and behavior. J Cancer Educ.

[13] Weinberg AD, Cooper HP, Mejia NI, Spiker CA (2004). Attitudes of primary care physicians and specialists about cancer clinical trials: a survey of Texas physicians. Tex Med..

[14] Bylund CL, Weiss ES, Michaels M, Patel S, D’Agostino TA, Peterson EB, Binz-Scharf MC, Blakeney N, McKee MD (2017). Primary care physicians' attitudes and beliefs about cancer clinical trials. Clin Trials.

[15] Michaels M, D'Agostino TA, Blakeney N, Weiss ES, Binz-Scharf MC, Golant M, Bylund CL (2015). Impact of primary care provider knowledge, attitudes, and beliefs about cancer clinical trials: implications for referral, education and advocacy. J Cancer Educ.

[16] Abraham J, Keresztury J, Azar J, Monga M, Bowers T, Jones MP, Tirona MT, Pollock J, Coonley C, Jubelirer S, Frame J, Fogg P, Getto M, Filburn S, Naim J, Lucas D, Petros W, Hall S, Remick SC (2009). Building a statewide clinical trials network for cancer care in West Virginia. W V Med J.

[17] Ellis SD, Geana M, Mackay CB, Moon DJ, Gills J, Zganjar A, Brekke G, Thrasher JB, Griebling TL (2019). Science in the heartland: exploring determinants of offering cancer clinical trials in rural-serving community urology practices. Urol Oncol.

[18] Friedman DB, Foster C, Bergeron CD, Tanner A, Kim SH (2015). A qualitative study of recruitment barriers, motivators, and community-based strategies for increasing clinical trials participation among rural and urban populations. Am J Health Promot.

[19] Michaels M, Weiss ES, Guidry JA, Blakeney N, Swords L, Gibbs B, Yeun S, Rytkonen B, Goodman R, Jarama SL, Greene AL (2012). The promise of community-based advocacy and education efforts for increasing cancer clinical trials accrual. J Cancer Educ.

[20] Kenny A, Hyett N, Sawtell J, Dickson-Swift V, Farmer J, O'Meara P (2013). Community participation in rural health: a scoping review. BMC Health Serv Res.

[21] Levit LA, Byatt L, Lyss AP, Paskett ED, Levit K, Kirkwood K, Schenkel C, Schilsky RL (2020). Closing the rural cancer care gap: three institutional approaches. JCO Oncol Pract.

[22] Chandra A, Miller CE, Acosta JD, Weilant S, Trujillo M, Plough A (2016). Drivers Of Health As A Shared Value: Mindset, Expectations, Sense Of Community. And Civic Engagement Health Aff (Millwood).

[23] Florin D, Dixon J (2004). Public involvement in health care. BMJ.

[24] Morgan LM (2001). Community participation in health: perpetual allure, persistent challenge. Health Policy Plan.

[25] County Health Rankings. https://www.countyhealthrankings.org/take-action-to-improve-health/partner-center/community-members#:~:text=Community%20members%20are%20context%20experts%20--%20they%20can,and%20networkers.%20It%E2%80%99s%20critical%20to%20value%20their%20expertise. Accessed 5 June 2023.

[26] Dorgan KA, Hutson SP, Gerding G, Duvall KL (2009). Culturally tailored cancer communication, education, and research: the highways and back roads of Appalachia. Prev Chronic Dis.

[27] Ko LK, Scarinci IC, Bouchard EG, Drake BF, Rodriguez EM, Chen MS, Kepka D, Kruse-Diehr AJ, Befort C, Shannon J, Farris PE (2022). A framework for equitable partnerships to promote cancer prevention and control in rural settings. JNCI Cancer Spectr.

[28] Vanderpool RC, Gainor SJ, Conn ME, Spencer C, Allen AR, Kennedy S (2011). Adapting and implementing evidence-based cancer education interventions in rural Appalachia: real world experiences and challenges. Rural Remote Health.

[29] Fernandez ME, Ten Hoor GA, Van Lieshout S, Rodriguez SA, Beidas RS, Parcel G, Ruiter RA, Markham CM, Kok G (2019). Implementation mapping: using intervention mapping to develop implementation strategies. Front Public Health.

[30] Damschroder LJ, Aron DC, Keith RE, Kirsh SR, Alexander JA, Lowery JC (2009). Fostering implementation of health services research findings into practice: a consolidated framework for advancing implementation science. Implement Sci.

[31] CFIR, Domains. CFIR Guide. 2009. https://cfirguide.org/guide/app/#/guide_select. Accessed 5 Jun 2023.

[32] CFIR, Templates Ta. CFIR Guide. 2009. https://cfirguide.org/guide/app/#/guide_select. Accessed 5 Jun 2023.

[33] Gürel E (2017). SWOT analysis: a theoretical review. J Int Soc Res.

[34] Pestle analysis. What Is PESTLE Analysis? A Tool for Business Analysis. PESTLE Analysis. 2011. https://pestleanalysis.com/what-is-pestle-analysis/. Accessed 15 Oct 2021.

[35] Kok G, Gottlieb NH, Peters GJ, Mullen PD, Parcel GS, Ruiter RA, Fernández ME, Markham C, Bartholomew LK (2016). A taxonomy of behaviour change methods: an Intervention Mapping approach. Health Psychol Rev.

[36] Koutoukidis DA, Lopes S, Atkins L, Croker H, Knobf MT, Lanceley A, Beeken RJ (2018). Use of intervention mapping to adapt a health behavior change intervention for endometrial cancer survivors: the shape-up following cancer treatment program. BMC Public Health.

[37] Paskett ED, Cooper MR, Stark N, Ricketts TC, Tropman S, Hatzell T, Aldrich T, Atkins J (2002). Clinical trial enrollment of rural patients with cancer. Cancer Pract.

