# Supplementary material for: Rural research capacity: a co-created model for research success
Source: Health Res Policy Syst. 2023 Jul 24;21:76. doi: 10.1186/s12961-023-01030-5 (PMC10364434; doi:10.1186/s12961-023-01030-5)
Supplement: Supplementary file 1 — Additional file 1: Appendices: Interview guide and ROCR system products. [file 12961_2023_1030_MOESM1_ESM.pdf]

APPENDICES

Rural Research Capacity Interview Questions

Regional Cultural Landscape

Researcher Request to Community form

Regional Capacity Response form

Rural Research Capacity Study Interview Questions

Pulled from: https://cfirguide.org/guide/app/#/guide_select

This first interview is meant to assess for assets or needs to run cancer prevention and control research studies as well as to identify adopters and implementers of these studies; people who would be considered stakeholders of the effort to implement cancer prevention and control research studies.

ASSET ASSESSMENT

COMPATIBILITY:

1. How well do cancer prevention & control research studies fit with existing work processes and practices in your setting?
   a. What are likely issues or complications that may arise?
2. Can you describe how cancer prevention & control research studies will be integrated into current processes?
   a. How will it interact or conflict with current programs or processes?
3. Will cancer prevention & control research studies replace or compliment a current program or process?
   a. In what ways?

RELATIVE PRIORITY:

4. To what extent might the implementation take a backseat to other high-priority initiatives going on now?
   a. How important do you think it is to implement cancer prevention & control research studies compared to the other priorities?
   b. How important is it to others, such as your coworkers or leaders, to implement cancer prevention & control research studies compared to the other priorities?

INNER SETTING – STRUCTURAL CHARACTERISTICS:

5. How will the infrastructure of your organization (social architecture, age, maturity, size, or physical layout) affect implementation of cancer prevention & control research studies?
   a. How will the infrastructure facilitate/hinder implementation of cancer prevention & control research studies?
   b. DO NOT ASK RIGHT NOW: How will you work around structural challenges?
6. What kinds of infrastructure changes will be needed to accommodate cancer prevention & control research studies?
   a. Changes in scope of practice? Changes in formal policies? Changes in information systems or electronic records systems? Other?
   b. What kind of approvals will be needed? Who will need to be involved?

CULTURE:

7. How would you describe the culture of your organization? Of your own setting or unit?
   a. Do you feel like the culture of your own unit is different from the overall organization? In what ways?
8. How do you think your organization's culture (general beliefs, values, assumptions that people embrace) will affect the implementation of cancer prevention & control research studies?
   a. Can you describe an example that highlights this?

COSMOPOLITANISM; ENCOURAGEMENT TO BRING NEW IDEAS TO ORG:

9. When you think of other organizations throughout Coos and Curry counties, which ones come to mind in terms of trying new ideas?
10. Which ones come to mind in terms of encouraging networking with colleagues outside their own settings?
11. To what extent does your organization encourage you to network with colleagues outside your own setting?
    a. Are you able to attend local/national conferences? Other opportunities?

PEER PRESSURE:

12. Can you tell me what you know about any other organizations that have implemented cancer prevention & control research studies or other similar programs?
13. To what extent would implementing cancer prevention & control research studies provide an advantage for your organization compared to other organizations in your area?
    a. Is there a competitive advantage?
    b. Is there something about cancer prevention & control research studies that would bring more individuals into your organization, instead of another one in your area?

<u>IDENTIFICATION OF ADOPTERS/ IMPLEMENTERS; INTERNAL & EXTERNAL</u>

TENSION FOR CHANGE:

14. Is there a strong need for cancer prevention & control research studies?
    a. Why or why not?
    b. Do others see a need for cancer prevention & control research studies?
15. How do people feel about current programs/practices/process that are available related to cancer prevention & control research studies?
16. To what extent do current programs fail to meet existing needs? Will cancer prevention & control research studies meet these needs?
17. How will cancer prevention & control research studies fill current gaps?

IMPLEMENTATION CLIMATE:

18. What is the general level of receptivity in your organization to implementing cancer prevention & control research studies?
    a. Why?

PROCESS – ENGAGING OPINION LEADERS:

19. Who are the key influential individuals to get on board with implementation of cancer prevention & control research studies?

CHAMPIONS:

20. Other than the formal implementation leader, are there people in your organization who are likely to champion (go above and beyond what might be expected) cancer prevention & control research studies?

PATIENT NEEDS/ RESOURCES:

1. To what extent is staff aware of the needs and preferences of the individuals being served by your organization?
    a. How "in touch" are staff and leadership with the individuals served by your organization?
2. How well do you think cancer prevention & control research studies will meet the needs of the individuals served by your organization?
    a. In what ways will cancer prevention & control research studies meet their needs?
    b. PROBES, if needed: E.g. improved access to services? Reduced wait times? Help with self-management? Reduced travel time and expense?

3. How do you think individuals served by your organization will respond to cancer prevention & control research studies?
4. What **barriers** will the individuals served by your organization face to participating in cancer prevention & control research studies?
5. Have you heard stories about the experiences of participants who participate in cancer prevention & control research studies?
    a. Can you describe a specific story?

coos & curry counties cultural landscape 2021

Researcher Resource: A description about this region of interest meant to help guide design choices for proposed cancer prevention and control projects and/or clinical trial research implementation.

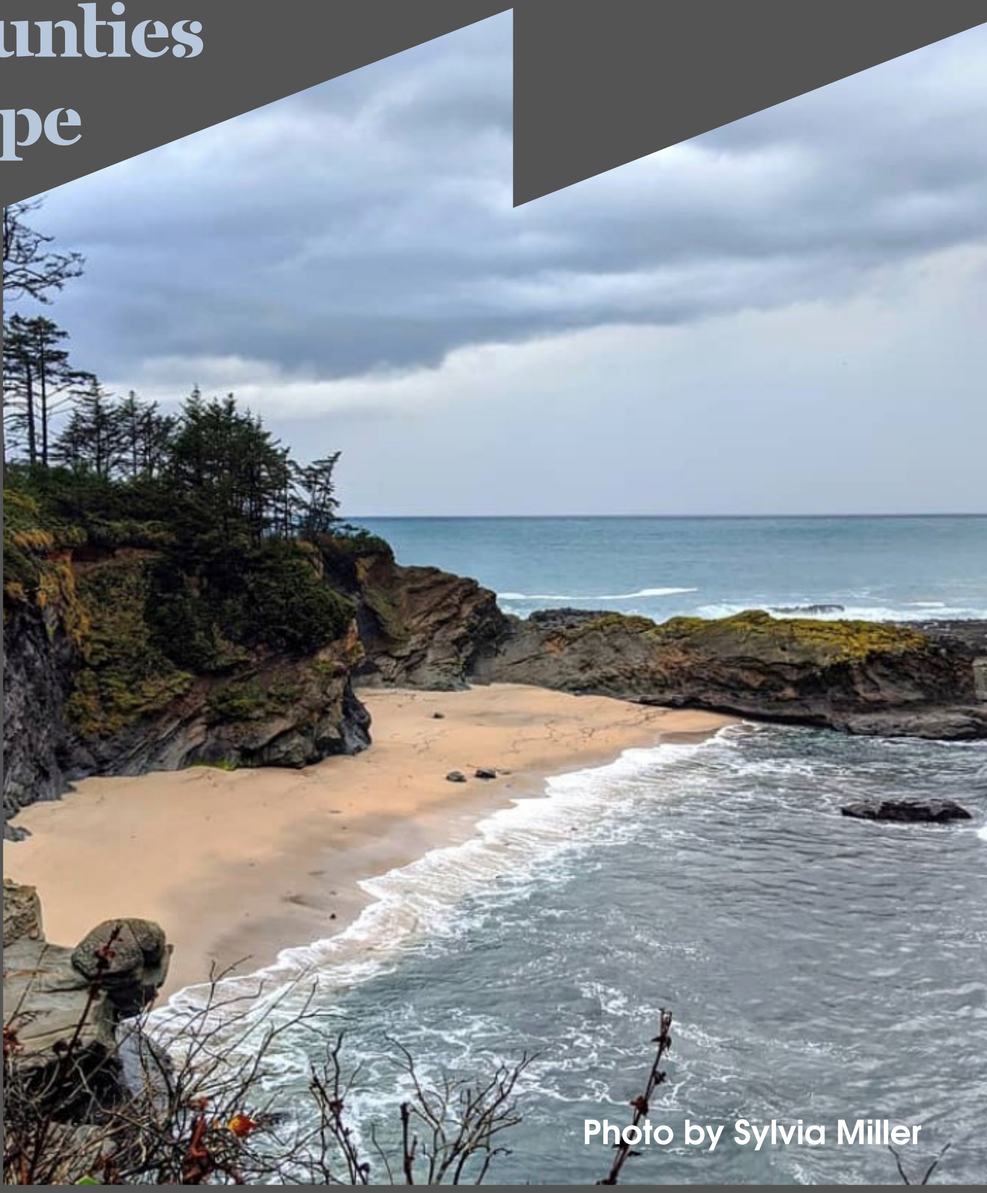

Photo by Sylvia Miller

cancer*

(*mortality rates are deaths per 100,000 residents; citations on page 2.)

lung & bronchus cancer

2nd highest cancer diagnosis at Bay Area Hospital. Women in Coos and Curry have the 3rd & 2nd highest lung and bronchus cancer incidence rates. Bay Area Hospital has an annual low-dose CT scan program available to screen and detect this cancer early.

colorectal cancer (crc)

Coos & Curry have much higher mortality of CRC than incidence rates. 34 Douglas County residents are diagnosed with CRC per year. Women in Coos have the highest mortality rate in Oregon. At home stool collection kits can detect this cancer early.

oropharyngeal cancer (back of throat, base of tongue/tonsils)

- Coos has the 2nd highest mortality rate of oropharyngeal cancer in Oregon. In 2018, 58% of these cancers diagnosed at Bay Area Hospital were stage III or IV (late stage).
- Tobacco and/or use has, historically, been the primary cause of oropharyngeal cancers.
- Human Papillomavirus infection is thought to be responsible for ~70% of all oropharyngeal cancer diagnoses. (https://www.cdc.gov/cancer/hpv/statistics/index.htm)

Residents of the South Coast die of cancer at a higher rate than the rest of the state (Coos: 197.6 - Curry: 182.7 - Douglas: 187.7 - Oregon: 162.8). Rural communities with low incidence rates and high mortality rates often reflect gaps in early detection, effective screening programs and/or fear that a cancer diagnosis is a death sentence.

cancer prevention and modifiable risk factors

- Coos County has a lower percentage of the population participating in preventative screenings than Oregon, which is an indicator of access to care, quality and timeliness of care (from Coos' Community Health Assessment).
- South Coast residents are about 4-10% more obese than rest of state (Coos: 38.3% - Curry: 33.4% - Douglas: 32.7% - Oregon: 28.6%).
- Coos, Curry & Douglas Counties all rank in the state's top 10 for percentage of residents with Chronic Lower Respiratory Diseases and Cardiovascular Disease.
- Risk factors for pancreatic cancer:
  - Tobacco usage: Coos County smoking rates are 2nd highest in state, Curry smoking rates are 4th highest.
  - Diabetes: Coos rates 7th highest, Douglas rates 6th highest.

economic

- **Homelessness: 1,299 homeless individuals (2019), a 36% increase from 2018.**
- In the 2016-2017 school year, 559 children were homeless, 52 of those students were pre-kindergarten ages.
- 1% housing availability in Coos; 72% of vacant housing used for vacation rentals in Curry.
- **37% of households in Coos County & 35% of households in Curry County are cost burdened, meaning their rent or mortgage exceeds 30% of their household income, this is higher than state levels.**
- High unemployment rate: 6.9% (2017) - Poverty rate: 21.7% (2019).
- Low Socio-Economic Status, median & average incomes are lower than state average. Difference in income by race/ethnicity is lower than state average.
- 100% Coos & Curry County students receive free/reduced-price lunches due to the COVID-19 pandemic (50% pre-pandemic).
- **Major Employers: Correctional Institutes in Coos & Curry. Coos County Shutter Creek to close in 2022.**

social

- Lack of childcare, resources, and education.
- Teen pregnancy rates, Coos: 13.1 per 1,000 (2011-2017 Oregon Health Authority)
- High school graduation rates, 2016: Coos-58% | Curry-72.6%
- Broadband access is limited - library parking lots were used to access free Wi-Fi during the COVID-19 pandemic.
- Census 2020 Rates:
  - **Coos County: 82.5% White/NHL, 6.6% Hispanic or Latino, 2% American Indian**
  - **Curry County: 82.9% White/NHL, 7.1% Hispanic or Latino, 2% American Indian**
  - **Douglas County: 84.1% White/NHL, 6% Hispanic or Latino, 1.5% American Indian**
- Those <65 with disabilities: Coos-16.4% | Curry-13.6% | Douglas-13.5% | state-16.8%
- Veterans in the Region: Coos-6,899 | Curry-2,714 | Douglas-12,354
- Seniors >65: Coos-26.5% | Curry-35% | Douglas-26.3%
  - Number of senior centers and clubs in North Bend/Coos Bay, including Veterans of Foreign Wars, Elks, Rotary, Lions Club.

healthy options and health care access

- Lack of health consciousness/lifestyle, which impacts health, leading to high incidence of health problems.
- Consumption of unhealthy foods, including soda, is higher in Coos County.
- **A third of the population in Coos & Curry Counties live in a food desert (both have a food environment index score of 6.9). Access to fresh produce is limited (both financially and geographically).**
- Lack of larger community gathering space(s), no accessible community center.
- Prescribing patterns for Medicare enrollees in Coos County in 2013-2014 show higher rates of opioid prescriptions than state and national trends (Center for Medicare and Medicaid Services).
- **100% of people in Coos County are considered to be in a health service shortage area (CHA and Office of Rural Health designation; also a recruitment and retention issue).**
- Physical fitness and recreation facilities are at a lower rate than the state, although there are Senior Centers in Coos Bay and North Bend. There are few sidewalks and bike lanes available for recreation along roads.
- Local physicians have a great deal of autonomy, so it can be challenging to coordinate care across offices.
- "...apparently, the thing you do after you get a doctor here is find another because your doctor's going to be leaving soon." -Research Interview Participant

political

- Community members and healthcare providers are fiercely independent.
- **Mistrust of Government organizations as well as mistrust or tension regarding "outsiders" (even from Portland area); resistance to outside involvement.**
- People who move in from other communities can create a divide.
- External impacts on the region: Lack of those who value rural communities, impacting recruitment & retention.

environmental

- **Limited access to transportation (distance, driving ability, car ownership) as well as limited public transit.**
- Fire season can drastically affect air quality (has been true for Curry, potential for Coos), although air quality is great when there are no fires.
- Year-round recreation due to climate; mountain bike trails recently built (youth involved) and grant funding to expand; beaches/ocean may be leveraged- connected to nature, moving, hiking, physical activity.

Thank you, researcher, for taking the time to orient yourself to Coos and Curry Counties via this locally-developed Cultural Landscape. Data collected in response to the development of this document was offered by community leaders in the region; specifically those who volunteered to participate on a research project to develop a community-involved research review process. This regionally-specific Cultural Landscape is the first step of the *Research in Oregon Communities' Review System* supported by OHSU's Community Outreach, Research and Engagement team.

Date finalized: July 2021 - Census updates: May 2022

National Cancer Institute (2021) State Cancer Profiles: Death Rates Table. National Vital Statistics System,2014-2018. http://www.https://statecancerprofiles.cancer.gov/deathrates/index.php (Accessed January 2021).

National Program of Cancer Registries SEER*Stat Database (2001-2017). United States Department ofHealth and Human Services, Centers for Disease Control and Prevention (based on the 2019 submission). http://www.https://statecancerprofiles.cancer.gov/incidencerates/index.php (Accessed January 2021).

Oregon Behavioral Risk Factor Surveillance System County Combined dataset, 2014 –2017. Oregon HealthAuthority. Health Promotion and Chronic Disease Prevention Section. Accessed January 2021.

Researcher Request to Community Form

Q1.1 Please select the region/county where you want to conduct your research/project:

Q1.3 Please select all counties that are included in your full catchment area:

Q1.4 **Project information -** Please provide the following:

Requesters Name: (1) ____

Project Lead Name: (2) ____

Project Title: (7) ____

Organization: (3) ____

Collaborating Organization (if any): (4) ____

E-mail: (5) ____

Phone: (6) ____

Q1.5 Is the project funded?

Yes. Please list funder name: (1)

Pending (2)

No (3)

Q2.1 Please tell us how much time you intend to spend in the region in relation to the conduct of your project. Tell us how much time your staff intend to spend in the region to conduct the project.

Q2.2 More specifically, how available are you (researcher), for instance, when confronted with a problem to solve in the region? (Note: communities will prefer that you are 'easily accessible'.)

Q2.3 If your availability is limited, are you willing to 1) collaboratively engage and include a community investigator or 2) offer to include another similar 'position' in the conduct or analysis of the project?
If YES, tell us how you would like people in the region to be engaged in conducting any part of your project?

Yes. Please indicate how. (1) ____

No (2)

Q2.4 What is your project's topic of interest? (e.g., nutrition, physical activity, survivorship, early detection of cancer, screening, etc.)

Q2.5 In lay language, provide the aims of your project. Tell us the objectives and any disease focus of your research/ project/ initiative.

Q2.6 At what stage of the research process are you in? (e.g. Are you still designing your project? Have you already received funding? Are you facing recruitment problems? Something else?)

Q2.7 Are you willing to consider suggestions or modifications if the regional community advisory deems the protocol not feasible as written/ designed (especially in relation to the advisory's region, community, population of interest, or partners)?

Yes (1)

Maybe (2)

No (3)

Q2.8 What do you intend to do with your results? Please consider the fact that if the community is interested in your project, they will also want to learn the results.

Q2.9 Will coordination with other departments, services, community organizations be required for study visits or procedures? Is your project specific to disease type?

If YES to either question, tell us what services or resources you envision needing to successfully conduct your project.

Q2.10 What is the scope of your project? Meaning; is this a project that can recruit from the community as a whole or does it need to be implemented in collaboration with a specific organization?

Q2.11 Through whose IRB will (or must) your project be submitted and reviewed?

Q2.12 Will training be provided?

Q2.13 Does your organization have a Diversity Equity Inclusion (DEI) framework that you run your work through? How have you considered or addressed the following for your project:

What categories of gender/sex are you collecting? For what purpose?
How will you respectfully address participants' identities and use their terms?
How does your project accommodate participants whose primarily language is not English?
How have you made your project accessible to disabled populations?

Q3.1 **Section 2. The following questions pertain to your project participants.**

Q3.2 Please describe your target population. What specific characteristics of your participants should we know about? What are your inclusion and exclusion criteria (the community will be assessing how restrictive or narrow they might be)?

Q3.3 What population age range are you targeting?

Youngest (1) ____

Oldest (2) ____

Q3.4 What barriers to care or research participation do you already know that your specific target population experiences? How have you accommodated known barriers in order to improve participation rates in previous projects?

Q4.1 **Section 3. The following questions pertain to the analysis and final product of your project.**

Q4.2 What impact to the community will your project have? People do not necessarily know that 'the research, outcomes or project' are for them. Please consider the following in your response:

What will you do with this data?
What do you want to learn?
What do you hope to accomplish through this project? Health outcomes?
Preliminary data for future grants?
At this stage of the process, what is your vision for ongoing impact once your project is complete?
What is the practical, on-the-ground application of your project and its outcomes? Sustainability:
How are you going to communicate your project's results to every day citizens to make the information useful or actionable?
How do you intend to involve the full community in the project (i.e., that it matters to them and the work is FOR them)?

Q4.3 What type of data do you want to collect?

Quantitative - data expressing a certain quantity, amount, or range (1)

Qualitative - non-numerical data that approximates and characterizes something (2)

Both quantitative and qualitative data (3)

Q4.4 What is your proposed method for collecting data for your project? (Select all that apply.)

Interviews (1)

Conversation Cafe-type opportunities (2)

Focus groups (3)

Electronic survey (4)

Telephone survey (5)

Paper survey (6)

Biological samples (7)

Biometric data (8)

Chart scrub via Epic (9)

Chart scrub via other EMRs (10)

Other (11) ____

Q4.5 Please describe how you are planning to handle and/or analyze this data.

Q4.6 Do you have stakeholders who are invested in the results of this project?

Yes. Please list who the invested stakeholders are: (1)

No (2)

Q4.7 Are the stakeholders going to act on your project's result?

Yes (1)

No (2)

Q4.8 What is the final product of this project?

Regional Capacity Response form

Q1 Region/county

- ○ Click to write Choice 1 (1)
- ○ Click to write Choice 2 (2)
- ○ Click to write Choice 3 (3)

Q2 **Project information -** Please provide the following:

- ○ Requesters Name: (1) ____
- ○ Project Lead Name: (2) ____
- ○ Project Title: (3) ____

Q3 **1. Population of Interest**

Q4 Does the region have access to the right patient population? Does the region feel the inclusion/exclusion criteria overly restrictive and narrow? (think about census data, cultural landscape, regional health improvement plan, etc.)

____

____

____

____

____

Q5 Which group of vulnerable populations are impacted?
What components of diversity, equity and inclusion are included in the design of the project?

Friendly for Spanish speaking population, disabled populations will be important/necessary. Does your org have a DEI framework that you (the researcher) run your work through? What categories of gender/sex are you collecting/ for what purpose? How will you respectfully use the terms the patient prefers?

____

____

Q6 Does the region think the proposed enrollment goal is realistic? Is the goal too high or too low?

____

Q31 Does the region think the amount of time to enroll community members realistic or sufficient? If not, how much time do we think we need?

____

Q7 Will enrollment compete with other studies/ efforts/ initiatives seeking the same patients? (Duplication of regional efforts.)

____

Q8 Are vulnerable populations involved (e.g., children, impaired adults) with special consent issues?

____

Q9 How will the subjects benefit from participating in the study?

____

Q10 Are patient compliance problems likely? If so, will it be necessary to monitor subjects' compliance with time-consuming phone calls or postcards? Is each touchpoint compensated/ sustainable to keep people engaged?

____

Q11 How might subjects be harmed (sensitive information disclosed or requested)? (e.g. trauma, habits, etc.)

____

Q12 **2. Project Plan or Protocol**
Q13 Is the protocol well designed and ethical?

____

Q14 How does this research align with the Oregon state improvement plan, local health improvement plan as well as the local public health and hospital health priorities? And how does this research align with the community's values?

____

Q15 Will people in this region be engaged in (conducting any part of) the project?

- ○ Yes. How so? (1) ____
- ○ No (2)

Q16 Will coordination with other departments, services, community organizations be required for study visits or procedures? How much coordination will be necessary?

____

Q17 Is necessary equipment available in the region?

____

Q18 (IF NECESSARY) Will the local IRB have problems with it? Is the protocol in final form? If not, how many amendments can be expected before it is in final form? Are case report forms complex?

____

Q19 What is the consent process proposed? Verbal? Written? Implied?

- ○ Verbal (1)
- ○ Written (2)
- ○ Implied (3)
- ○ Other: (4) ____

Q20 What are the plans for result dissemination to the community, participants, county, involved institutions, etc.? How are results of the study actionable in our community? Timeline for communicating with patients, community, the research advisory?

____

Q21 **3. Project Data Collection Procedures**
Q22 Are project or data collection procedures:

- ○ Frequent? (1) ____
- ○ Difficult? (2) ____
- ○ Time consuming? (3) ____
- ○ Accessible to all targeted participants? (4) ____
- ○ Inconvenient or multiple? (think about internet access, transportation, causing subjects to miss work/school, etc.) (5) ____
- ○ Compensated? (6) ____

Q23 **4. Staff Engaged in the Project from the region**
Q24 Is qualified staff available?

____

Q25 If needed, is researcher providing training to staff?

____

Q26 Can the workload of a new project fit into current workflow processes?

____

Q27 **5. Budget and/or Resource Needs**
Q28 What does the researcher's budget cover to support the project in our region? (e.g. incentives, training, staff support, transportation, office space required for recruitment/enrollment, etc.)

Review and consider your regional landscape and local challenges to assess.

____

Q29 From where does the researcher expect to recruit patients? If so, is there adequate clinic/office space available? And will the researcher provide funding for its access?

____

Q30 If not paying for full-time support staff or things like transportation, will researcher pay for processes or items that the regional community advisory knows will lead to successful implementation of the project, such as:

- ○ Negotiating with additional community organizations to support the project? (1) ____

- ○ Paying for promotional and targeted messages? (2) ____

- ○ Translation of materials into Spanish and other languages? (3) ____

- ○ How many organizations do we know it will take to reach recruitment numbers? (4) ____

- ○ How many organizations have expressed interest in this project – and therefore, is each organization supported? (5) ____

- ○ Assure/ confirm non-duplication of efforts between involved organizations. (6) ____

- ○ Source of the project funds (e.g. Marlboro) to assess conflicts of interest. (7) ____
